# Consensus on the use of ropeginterferon alfa-2b in Japanese patients with polycythemia vera: a modified Delphi survey

**DOI:** 10.1007/s12185-025-04140-8

**Published:** 2026-03-04

**Authors:** Kazuya Shimoda, Katsuto Takenaka, Haruna Kitamura, Koji Dohi, Riki Yoshimoto, Keita Kirito

**Affiliations:** 1 Division of Hematology, Diabetes and Endocrinology, Department of Internal Medicine, University of Miyazaki, 5200 Kihara, Kiyotakecho, Miyazaki-shi, Miyazaki 889-1692 Japan; 2 Department of Hematology, Clinical Immunology and Infectious Diseases, Ehime University Graduate School of Medicine, Toon, Ehime Japan; 3 LESPEDEZA, EMC K.K., Tokyo, Japan; 4 Department of Hematology and Medical Oncology, Yamanashi University School of Medicine, Chuo-shi, Yamanashi Japan

**Keywords:** Cytoreductive therapy, Delphi, Polycythemia vera, Ropeginterferon alfa-2b

## Abstract

Polycythemia vera (PV) is a myeloproliferative neoplasm that is treated according to the patient’s thrombosis risk, including cytoreductive therapies for those at high-risk for thrombosis. Ropeginterferon alfa-2b, a mono-pegylated interferon α-2b, is a new cytoreductive therapy for PV. This expert consensus study, conducted before ropeginterferon alfa-2b recommendations were added to the Japanese guidelines, used a four-phase Delphi method to survey 18 Japanese hematology experts and develop suggestions for ropeginterferon alfa-2b use in Japan. Several scenarios where ropeginterferon alfa-2b treatment is considered appropriate for patients with PV were identified, including: 1) low-risk patients requiring cytoreductive therapy (excluding low-risk patients requiring cytoreductive therapy due to splenomegaly); 2) patients intolerant or resistant to hydroxyurea treatment; 3) high-risk patients aged ≤ 70 years, even if hematocrit < 45% is achieved and maintained under hydroxyurea treatment; and 4) patients who wish to conceive or who are pregnant and require cytoreductive therapy. This is the first expert consensus study using the Delphi method to examine cytoreductive therapy for Japanese patients with PV and contributes to the appropriate selection of patients for ropeginterferon alfa-2b in clinical practice.

**Supplementary Information:**

The online version contains supplementary material available at 10.1007/s12185-025-04140-8.

## Introduction

Polycythemia vera (PV) is a Philadelphia chromosome-negative myeloproliferative neoplasm [1]. In patients with PV, the autonomous activation of the Janus kinase (JAK)/signal transducer and activator of transcription signaling cascade, primarily because of mutations in the *JAK2* gene [1–4], induces excessive production of three types of hematopoietic lineage cells, including red blood cells, granulocytes, and megakaryocytes. PV can transform into myelofibrosis or acute myeloid leukemia, and the presence of thrombocytosis and leukocytosis in patients with PV can increase the risk of hemorrhagic and thrombotic events [5].

The Japanese guidelines recommend treatment of PV according to the patient’s risk of thrombosis to prevent complicated thrombosis [6], with patients aged < 60 years without a history of thrombosis categorized as “low-risk PV” and patients aged ≥ 60 years and/or those with a history of thrombosis categorized as “high-risk PV”. Phlebotomy and low-dose aspirin are recommended for patients with low-risk PV [6]; cytoreductive therapies, in addition to phlebotomy and aspirin, are recommended for patients with high-risk PV [6].

Ropeginterferon alfa-2b is a new mono-pegylated interferon alfa-2b approved for PV in Europe and the United States [7, 8]. In March 2023, ropeginterferon alfa-2b was approved in Japan for the treatment of PV when the existing treatment is insufficient or inappropriate, based on the results of clinical studies conducted overseas and in Japan [9–11]. In December 2024, the Japanese guidelines were updated to state that treatment with cytoreductive therapy may be considered for patients with low-risk PV who have difficulty undergoing phlebotomy, markedly increased platelet and white blood cell counts, or splenomegaly, and that ropeginterferon alfa-2b may be used as the first-line treatment option [6]. For high-risk PV, the Japanese guidelines recommend hydroxyurea and ropeginterferon alfa-2b as first-line cytoreductive therapies [6]. Other guidelines (such as the European LeukemiaNet guidelines and guidelines for cancer treatment in the United States) also propose ropeginterferon alfa-2b as a treatment option for patients with low- and high-risk PV [12].

We performed an expert consensus study by surveying hematology experts in Japan to obtain their personal opinions, to develop suggestions for the appropriate use of ropeginterferon alfa-2b in patients with PV in Japan, using the Delphi method. The Delphi method is a consensus-building method that is often used for the preparation of guidelines; it concentrates reliable collective opinions via multiple rounds of anonymous feedback from experts, allowing participants to share their individual views without influencing one another’s opinions. At the time of conducting this consensus study, the Japanese guidelines did not include recommendations regarding the use of ropeginterferon alfa-2b in patients with low- or high-risk PV. The findings of this expert consensus survey provide further insight into which patient groups may be appropriate candidates for ropeginterferon alfa-2b treatment in Japan.

## Materials and methods

### Study design and participants

This was a consensus study that used a four-phase Delphi method (1. project planning; 2. literature review and statement generation; 3. Delphi survey; and 4. report development), characterized by panelist anonymity during the voting/selecting/rating steps, multiple voting rounds, and feedback to panelists regarding the previous Questionnaire findings prior to voting on the subsequent Questionnaire. The study was approved by the ethical review board of Yamanashi University School of Medicine, Japan (approval number: R05779; approval date: 18 March 2024) and conducted in line with the ACCORD guidelines [13].

All meetings were conducted in Japanese. The Steering Committee convened four times and included the following three individuals with specific expertise in the administration of ropeginterferon alfa-2b and clinical experience in PV: Kazuya Shimoda (University of Miyazaki), Keita Kirito (Yamanashi University School of Medicine), and Katsuto Takenaka (Ehime University Graduate School of Medicine).

Clinicians eligible for the expert panel were required to have experience in managing PV (defined as five or more patients) or to be specialists affiliated with hematology departments in institutions that are part of the Japanese Society of Hematology Myeloproliferative Neoplasm Retrospective Study 2018 (JSH-MPN-R18) Executive Committee. In addition, they were required to have experience with administering ropeginterferon alfa-2b to at least one patient. Based on these inclusion criteria, potential candidates were identified from (i) physicians affiliated with the JSH-MPN-R18 study (a nationwide study on thrombosis in myeloproliferative neoplasms), (ii) physicians at institutions that had participated in a Japanese phase 2 clinical trial [11], and (iii) physicians who had previously used ropeginterferon alfa-2b in clinical trials or routine clinical practice. A list of 18 eligible candidates was compiled and reviewed by the Steering Committee, who confirmed the final panel members based on clinical expertise and prior study involvement.

The secretariat, who was not directly involved in key aspects of the study, sent invitation emails to the candidates. The candidates completed a self-attestation checklist for the selection criteria via a dedicated web page linked in the email. Formal eligibility was reconfirmed through this process so that only those who met the predefined criteria could proceed to provide an electronic informed consent (e-signature), while those who did not meet the criteria were shown an error message and were not able to continue. The informed consent form also clearly disclosed the sponsor of the study, and participation proceeded only after panelists confirmed their understanding of the potential benefits and disadvantages. No financial remuneration was provided to the panelists. Personally identifiable information was shared only with the secretariat’s data manager for operational purposes and was not accessible to the Steering Committee or authors outside the secretariat.

### Literature review

**Fig. 1:**
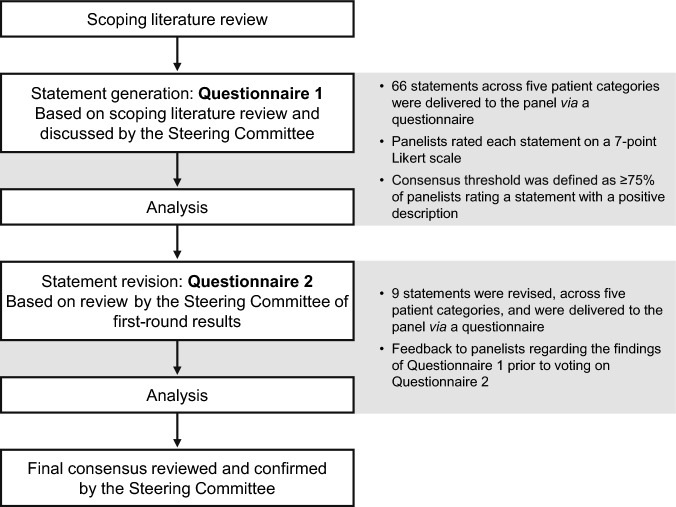
The review process. The process was based on the Delphi method

A flow chart depicting the process from literature review to formation of consensus is shown in Fig. 1. Prior to drafting the statements, a scoping literature review was conducted based on the following preliminary research questions: “In low-risk PV patients not receiving cytoreductive therapy (treated with aspirin and/or phlebotomy), when should cytoreductive therapy with ropeginterferon alfa-2b be added?”; “In high-risk PV patients who are a) treated with aspirin and/or phlebotomy; b) treated with hydroxyurea or ruxolitinib, when should cytoreductive therapy with ropeginterferon alfa-2b be initiated?”; and “What types of patients with low-risk or high-risk PV could potentially qualify for treatment with ropeginterferon alfa-2b?” The review was limited to publications indexed in PubMed (published between 1 January 2013 and 28 November 2023, including guidelines, reviews, and expert opinions and commentaries) and the Japanese database, Ichu-shi (published between 1 January 2013 and 18 October 2023, including original articles, commentaries, reviews, commentaries of illustrations, Questions & Answers, lectures, conference proceedings, and case reports). The following keywords were used: Polycythemia vera (Medical Subject Headings term, keyword, and synonyms) | low-risk | high-risk | *JAK2* (V617F); Ropeginterferon alfa-2b | cytoreductive (therapy); Hydroxyurea | Ruxolitinib; and Hemorrhag*/haemorrhag* | Bleed* | Thrombo* | Thromboemboli* | Splenomegal* | Symptom* | Morpholog* | Leukocyt* | Thrombocyt* | Cardiovascular (risk).

### Statement generation, panelist voting, and consensus

Draft statements were generated in Japanese by LESPEDEZA, EMC K.K., based on the results of the scoping literature review. The statements were discussed by the Steering Committee, and an initial set of 66 statements for inclusion in Questionnaire 1 was agreed upon (Fig. 1). The fixed statements were then translated into English (Supplementary Table 1).

Each statement was related to the following patient populations: patients with low-risk PV being treated with phlebotomy and/or aspirin (population 1), patients with low-risk PV being treated with hydroxyurea (population 2), patients with high-risk PV who are about to initiate cytoreductive therapy (population 3), patients with high-risk PV being treated with hydroxyurea or ruxolitinib (population 4), and patients with PV who are pregnant or planning to conceive (population 5).

A web-based system was used to conduct the survey and collate the results. The panelists provided responses to the statements via a seven-point Likert scale. If ≥ 75% of panelists selected a positive rating (somewhat agree, agree, or strongly agree), the statement was considered to have achieved consensus. In cases where there were two linked statements (e.g., 1-1 and 1-2), if the first statement did not achieve consensus but the second statement did, the second statement was treated as reference data.

The first web-based Questionnaire (Questionnaire 1) was conducted between 28 May 2024 and 9 June 2024, and the results were aggregated from 10 June 2024 to 20 June 2024. Following the completion of Questionnaire 1, the Steering Committee reviewed the collated first-round results and revised the statements for Questionnaire 2 (Fig. 1). The panelists were instructed to review the results of Questionnaire 1 prior to responding to Questionnaire 2 so that they could make judgments with awareness of the overall views of the panel prior to responding to Questionnaire 2. Panelists then provided responses to Questionnaire 2 from 6 August 2024 to 27 August 2024. These results were aggregated from 28 August 2024 to 18 September 2024.

## Results

### Panelists

The background information of the 18 expert panelists is shown in Table 1. All 18 panelists participated in both Questionnaire 1 and Questionnaire 2. Ten panelists (55.6%) had 20–29 years of clinical experience in hematology, and all panelists had cared for ≥ 10 patients with PV. Thirteen of the 18 panelists (72.2%) were hematology specialists at facilities affiliated with the JSH-MPN-R18 study. Most panelists (13/18, 72.2%) had previously treated one to four patients with ropeginterferon alfa-2b.

**Table 1:** Background characteristics of the panelists in the Delphi survey

	Panelists (N = 18)
Years of clinical experience in hematology	
≤ 19	3 (16.7)
20–29	10 (55.6)
30–39	5 (27.8)
Number of patients with PV that panelists have treated	
≥ 10	18 (100)
Hematological specialists in the facility to which JSH-MPN-R18 research executive committee members belong	
Yes	13 (72.2)
No	5 (27.8)
Number of patients treated with ropeginterferon alfa-2b	
1–4	13 (72.2)
5–9	1 (5.6)
≥ 10	4 (22.2)

Data are n (%)

*JSH-MPN-R18* Japanese Society of Hematology Myeloproliferative Neoplasm Retrospective Study 2018, *PV* polycythemia vera

### Overall agreement rates per patient group

**Fig. 2:**
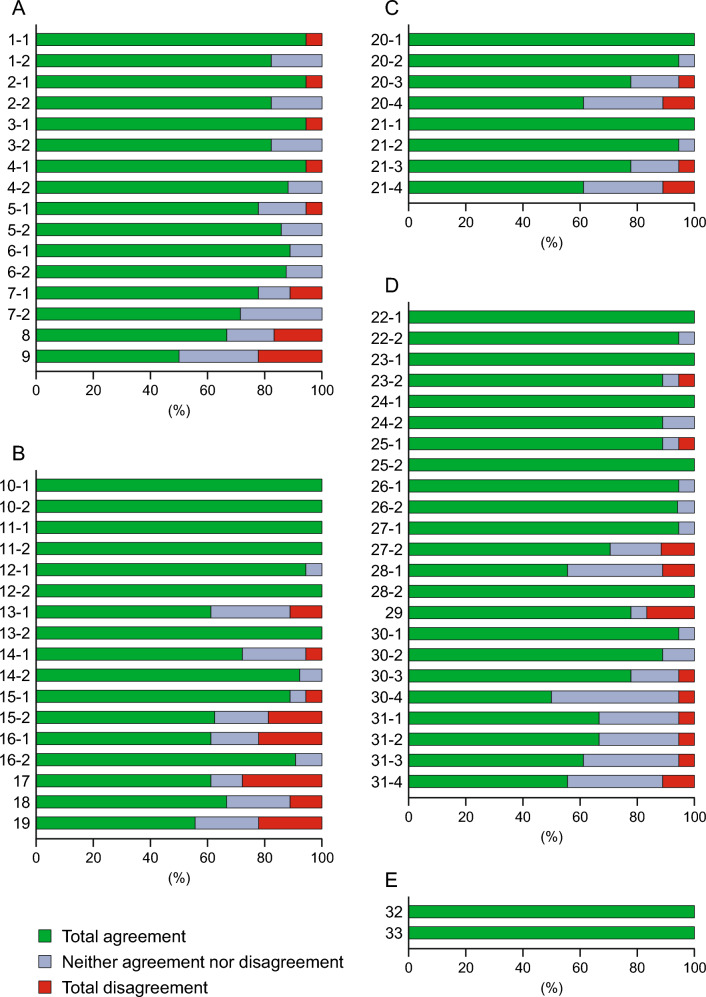
Level of agreement with statements in Questionnaire 2 for each patient category. Agreement for statements in **A** low-risk PV patients being treated with phlebotomy and/or aspirin (population 1), **B** low-risk PV patients being treated with hydroxyurea (population 2), **C** high-risk PV patients who are about to initiate cytoreductive therapy (population 3), **D** high-risk PV patients being treated with hydroxyurea or ruxolitinib (population 4), and **E** PV patients who are pregnant or planning to conceive (population 5). The y axis refers to the number of each statement, and the x axis shows the agreement rate. *PV* polycythemia vera

The content of each question in Questionnaires 1 and 2 and their agreement rates are shown in Supplementary Table 1. Additionally, Fig. 2 and Table 2 show the level of agreement with statements for each patient category and the summary of statements with consensus in Questionnaire 2, respectively.

**Table 2:** Summary of statements with consensus

**Patients with low-risk PV**				
Current treatment		Eligible patients	Initiate cytoreductive therapy?	Is ropeginterferon alfa-2b the drug of choice?
Under treatment with phlebotomy and/or aspirin	Under treatment with a Ht target of < 45% with phlebotomy	If syncope or blood phobia is observed following treatment with phlebotomy, or if intravenous access is difficult Table S1: statement 1-1	Yes Table S1: statements 1-1, 2-1, and 3-1	Yes Table S1: statements 1-2, 2-2, and 3-2
		If symptoms associated with iron deficiency (e.g., malaise) persist after treatment with phlebotomy Table S1: statement 2-1		
		If a patient feels pain resulting from phlebotomy Table S1: statement 3-1		
		If symptoms (e.g., itching, headache, erythromelalgia, or vasomotor symptoms not responsive to aspirin) are not improved by treatment with phlebotomy and/or aspirin Table S1: statement 4-1	Yes Table S1: statements 4-1, 5-1, and 6-1	Yes Table S1: statements 4-2, 5-2, and 6-2
		If significant thrombocytosis (platelet count > 1,000 × 10^9^/L) persists Table S1: statement 5-1		
		If leukocytosis (white blood cell count > 15 × 10^9^/L) persists Table S1: statement 6-1		
		If splenomegaly (with confirmation that there is no progression to myelofibrosis) is observed Table S1: statement 7-1	Yes Table S1: statement 7-1	No Table S1: statement 7-2

Current treatment		Eligible patients	Switch to another drug?	Is ropeginterferon alfa-2b the drug of choice?
Under treatment with hydroxyurea		If unacceptable non-hematological toxicity (e.g., leg ulcers, mucocutaneous manifestations, gastrointestinal symptoms, pneumonitis, or fever) occurs Table S1: statement 10-1	Yes Table S1: statements 10-1 and 11-1	Yes Table S1: statements 10-2 and 11-2
		If symptoms (e.g., itching, headache, erythromelalgia, or vasomotor symptoms not responsive to aspirin) are not improved Table S1: statement 11-1		
	At the maximum tolerated dose of hydroxyurea	If Ht < 45% cannot be achieved or frequent phlebotomy is necessary to achieve Ht < 45% Table S1: statement 12-1	Yes Table S1: statement 12-1	Yes Table S1: statement 12-2
		If a reduction in splenomegaly (with confirmation that there is no progression to myelofibrosis) is not observed Table S1: statement 15-1	Yes Table S1: statement 15-1	No Table S1: statement 15-2

**Patients with high-risk PV**				
Current treatment		Eligible patients	Initiate cytoreductive therapy?	Is ropeginterferon alfa-2b the first-line treatment?
About to initiate new cytoreductive therapy		Patients aged ≤ 70 years who are considered to require cytoreductive therapy Table S1: statements 20; 20-1 to 20-3, and 21; 21-1 to 21-3	Not Applicable	Yes Table S1: statements 20; 20-1 to 20-3, and 21; 21-1 to 21-3

Current treatment		Eligible patients	Switch to another drug?	Is ropeginterferon alfa-2b the drug of choice?
Under treatment with hydroxyurea		If unacceptable non-hematological toxicity (e.g., leg ulcers, mucocutaneous manifestations, gastrointestinal symptoms, pneumonitis, or fever) occurs Table S1: statement 22-1	Yes Table S1: statements 22-1 and 23-1	Yes Table S1: statements 22-2 and 23-2
		If symptoms (e.g., itching, headache, erythromelalgia, or vasomotor symptoms that are not responsive to aspirin) are not improved Table S1: statement 23-1		
	At the maximum tolerated dose of hydroxyurea	If Ht < 45% cannot be achieved or if frequent phlebotomy is necessary to achieve Ht < 45% Table S1: statement 24-1	Yes Table S1: statements 24-1, 25-1, and 26-1	Yes Table S1: statements 24-2, 25-2, and 26-2
		If thrombocytosis (platelet count > 400 × 10^9^/L) persists Table S1: statement 25-1		
		If leukocytosis (white blood cell count > 10 × 10^9^/L) persists Table S1: statement 26-1		
		If a reduction in splenomegaly (with confirmation that there is no progression to myelofibrosis) is not observed Table S1: statement 27-1	Yes Table S1: statement 27-1	No Table S1: statement 27-2

Current treatment		Eligible patients	Initiate cytoreductive therapy?	Switch to ropeginterferon alfa-2b?
Under treatment with hydroxyurea or ruxolitinib		If medication adherence is poor and blood cells are uncontrolled Table S1: statement 29	Not Applicable	Yes Table S1: statement 29
Under treatment with hydroxyurea		In patients ≤ 70 years, even if Ht can be controlled (Ht < 45% is achieved and maintained), treatment is switched to ropeginterferon alfa-2b based on a report showing that a decrease in *JAK2* V617F allele burden leads to an improvement in event-free survival Table S1: statements 30; 30-1 to 30-3	Not Applicable	Yes Table S1: statements 30; 30-1 to 30-3

**Patients with PV who are pregnant or planning to conceive**				
Current treatment		Eligible patients	Initiate cytoreductive therapy?	Is the use of ropeginterferon alfa-2b considered?
Initiate new cytoreductive therapy		In patients with PV who wish to conceive and require cytoreductive therapy Table S1: statement 32	Not Applicable	Yes Table S1: statement 32
		In patients with PV who are pregnant and require cytoreductive therapy Table S1: statement 33	Not Applicable	Yes Table S1: statement 33

*Ht* hematocrit, *JAK2* Janus kinase 2, *PV* polycythemia vera

In Questionnaire 1, 37/66 (56.1%) statements reached consensus. Statements relating to patients with low-risk PV being treated with phlebotomy and/or aspirin reached consensus for 11/16 (68.8%) statements; those relating to patients with low-risk PV being treated with hydroxyurea reached consensus for 7/17 (41.2%) statements; those relating to patients with high-risk PV who are about to initiate cytoreductive therapy reached consensus for 4/8 (50.0%) statements; those relating to patients with high-risk PV being treated with hydroxyurea or ruxolitinib reached consensus for 13/23 (56.5%) statements; and those relating to patients with PV who are pregnant or planning to conceive reached consensus for 2/2 (100%) statements.

Statements relating to splenomegaly, the *JAK2* V617F allele burden, high-risk patients aged ≥ 66 years, and drug adherence generally did not reach consensus in Questionnaire 1. Subsequently, nine statements were corrected and revised for inclusion in Questionnaire 2 (Supplementary Table 1).

In Questionnaire 2, 43/66 (65.2%) statements reached consensus. Statements relating to patients with low-risk PV being treated with phlebotomy and/or aspirin reached consensus for 13/16 (81.3%) statements; those relating to patients with low-risk PV being treated with hydroxyurea reached consensus for 7/17 (41.2%) statements; those relating to patients with high-risk PV who are about to initiate cytoreductive therapy reached consensus for 6/8 (75.0%) statements; those relating to patients with high-risk PV being treated with hydroxyurea or ruxolitinib reached consensus for 15/23 (65.2%) statements; and those relating to patients with PV who are pregnant or planning to conceive reached consensus for 2/2 (100%) statements. The rate of agreement in Questionnaire 2 was either maintained or increased compared with that in Questionnaire 1.

### Consensus reached

#### Population 1: Low-risk PV patients being treated with phlebotomy and/or aspirin

94.4% of the expert panelists agreed to initiate cytoreductive therapy if syncope or blood phobia is observed following treatment with phlebotomy, or if intravenous access is difficult (Statement 1-1), if symptoms associated with iron deficiency (e.g., malaise) persist after treatment with phlebotomy (Statement 2-1), or if a patient feels pain resulting from phlebotomy (Statement 3-1), and ≥ 80% of the expert panelists agreed that ropeginterferon alfa-2b should be started as the first-line cytoreductive treatment for these patients (Statements 1-2, 2-2, and 3-2).

If PV-related symptoms are not improved by treatment with phlebotomy and/or aspirin or if significant thrombocytosis (platelet count > 1,000 × 10^9^/L) or leukocytosis (white blood cell count > 15 × 10^9^/L) persists, 94.4%, 77.8%, and 88.9% of the expert panelists, respectively, agreed with initiating cytoreductive therapy (Statements 4-1, 5-1, and 6-1), and ≥ 85% of the panelists agreed with the use of ropeginterferon alfa-2b (Statements 4-2, 5-2, and 6-2). However, while the panel agreed that cytoreductive therapy should be initiated if splenomegaly (with confirmation that there is no progression to myelofibrosis) is observed (Statement 7-1), consensus was not achieved regarding the use of ropeginterferon alfa-2b as the first-line cytoreductive treatment (Statement 7-2). The expert panel also did not agree with statements regarding the use of ropeginterferon alfa-2b in patients with a high *JAK2* V617F allele burden (Statements 8 and 9).

#### Population 2: Low-risk PV patients being treated with hydroxyurea

If unacceptable non-hematological toxicity (e.g., leg ulcers, mucocutaneous manifestations, gastrointestinal symptoms, pneumonitis, or fever) occurs, or PV-related symptoms (e.g., itching, headache, erythromelalgia, or vasomotor symptoms) are not improved during treatment with hydroxyurea, all of the expert panelists agreed with switching to other drugs (Statements 10-1 and 11-1). If frequent phlebotomy is necessary during treatment with hydroxyurea at the maximum tolerated dose, most of the panelists (94.4%) agreed with switching to other drugs (Statement 12-1). 100% of panelists agreed with switching to ropeginterferon alfa-2b as the first-line treatment for these patients (Statements 10-2, 11-2, and 12-2). If a reduction in splenomegaly (with confirmation that there is no progression to myelofibrosis) is not observed during treatment with hydroxyurea at the maximum tolerated dose, 88.9% of the expert panel agreed with switching to other drugs (Statement 15-1), but consensus was not achieved regarding the use of ropeginterferon alfa-2b as the first-line treatment (Statement 15-2).

Consensus was not achieved regarding switching to other drugs if thrombocytosis (platelet count > 400 × 10^9^/L) or leukocytosis (white blood cell count > 10 × 10^9^/L) persists during treatment with hydroxyurea at the maximum tolerated dose (Statements 13-1 and 14-1). If the *JAK2* V617F allele burden value does not decrease during treatment with hydroxyurea at the maximum tolerated dose, consensus was not achieved regarding switching to other drugs (Statement 16-1).

Consensus was also not achieved regarding switching to ropeginterferon alfa-2b if blood cell control is insufficient because of poor medication adherence (Statement 17), if the risk of secondary leukemia is considered (Statement 18), or to decrease the *JAK2* V617F allele burden even if hematocrit can be controlled (Statement 19) during treatment with hydroxyurea.

#### Population 3: High-risk PV patients about to initiate cytoreductive therapy

Consensus was achieved regarding the use of ropeginterferon alfa-2b as the first-line treatment in high-risk PV patients aged ≤ 70 years about to initiate cytoreductive therapy (Statements 20; 20-1 to 20-3). However, consensus was not achieved regarding the use of ropeginterferon alfa-2b as the first-line cytoreductive treatment in high-risk patients aged > 70 years (Statement 20-4), even if the *JAK2* V617F allele burden value was > 50% at the time of diagnosis (Statement 21-4).

#### Population 4: High-risk PV patients being treated with hydroxyurea or ruxolitinib

For high-risk PV patients treated with hydroxyurea, all panelists agreed with switching to other drugs if unacceptable non-hematological toxicity occurs, if PV-related symptoms are not improved, persistent hematocrit values ≥ 45%, or requiring frequent phlebotomy (Statements 22-1, 23-1, and 24-1), and approximately 90% of the panelists agreed with switching to ropeginterferon alfa-2b (Statements 22-2, 23-2, and 24-2).

If thrombocytosis (platelet count > 400 × 10^9^/L) or leukocytosis (white blood cell count > 10 × 10^9^/L) persists during treatment with hydroxyurea at the maximum tolerated dose, most panelists (88.9% and 94.4%, respectively) agreed with switching to other drugs (Statements 25-1 and 26-1), and ≥ 94% of the panelists agreed with switching to ropeginterferon alfa-2b as the first-line treatment (Statements 25-2 and 26-2). If medication adherence is poor and blood cells are uncontrolled during treatment with hydroxyurea or ruxolitinib, 77.8% of panelists agreed that switching to ropeginterferon alfa-2b should be considered (Statement 29).

In patients aged ≤ 70 years, even if hematocrit can be controlled during treatment with hydroxyurea, ≥ 77% of panelists agreed that switching to ropeginterferon alfa-2b should be considered based on a report [14] showing that a decrease in the *JAK2* V617F allele burden leads to an improvement in event-free survival (Statements 30; 30-1 to 30-3). No consensus was achieved regarding switching to ropeginterferon alfa-2b in patients being treated with ruxolitinib, including those with controlled hematocrit based on that report (Statements 31; 31-1 to 31-4).

Consensus was achieved regarding switching to other drugs in cases where a reduction in splenomegaly (with confirmation that there is no progression to myelofibrosis) is not observed during treatment with hydroxyurea at the maximum tolerated dose (Statement 27-1), but no consensus was achieved regarding the use of ropeginterferon alfa-2b as the first-line treatment (Statement 27-2).

If the *JAK2* V617F allele burden value does not decrease during treatment with hydroxyurea, consensus was not achieved regarding switching to other drugs (Statement 28-1).

#### Population 5: PV patients who are pregnant or planning to conceive

In both Questionnaires 1 and 2, all the panelists agreed that patients with PV who are pregnant or planning to conceive and require cytoreductive therapy should be treated with ropeginterferon alfa-2b (Statements 32 and 33).

## Discussion

In this Delphi study, we collated and analyzed the consensus of Japanese hematology experts on the appropriate use of ropeginterferon alfa-2b in Japan. Several scenarios were identified in which treatment with ropeginterferon alfa-2b was considered appropriate in patients with low-risk PV treated with phlebotomy and/or aspirin. For low-risk PV patients with non-ameliorated symptoms or persistent marked thrombocytosis and/or leukocytosis, consensus was reached on the use of ropeginterferon alfa-2b as a first-line cytoreductive therapy in addition to phlebotomy. For high-risk PV patients about to initiate cytoreductive therapy and aged ≤ 70 years, consensus was reached on the use of ropeginterferon alfa-2b as a first-line cytoreductive treatment option. Consensus was also reached regarding switching to ropeginterferon alfa-2b in high-risk PV patients aged ≤ 70 years who were receiving hydroxyurea and had controlled hematocrit, based on a report that a decrease in the *JAK2* V617F allele burden levels may contribute to improved event-free survival [14]. Thus, many of the scenarios in which consensus was reached involved patients in whom standard therapies were inadequate, including those where patients experience adverse effects associated with standard therapies or where the response to treatment is insufficient.

In Questionnaire 2, the persistence of iron deficiency symptoms was considered sufficient to use ropeginterferon alfa-2b. Although the use of ropeginterferon alfa-2b is not specifically recommended, several clinical trials have confirmed the efficacy of ropeginterferon alfa-2b without causing iron deficiency [15, 16].

In patients with low-risk PV already being treated with hydroxyurea, switching to ropeginterferon alfa-2b was suggested for patients experiencing unacceptable non-hematological toxicity, persistent PV-related symptoms, or persistent hematocrit values ≥ 45%, or requiring frequent phlebotomy. Recent findings suggest that increased white blood cell counts and/or platelet counts are associated with an increased risk of thrombotic events in patients with PV [17, 18]. However, in Questionnaire 1, for patients with observed thrombocytosis or leukocytosis, only about half of the panelists agreed with the suggestion to switch to other drugs, and no consensus was reached. Reference values for platelet count (> 400 × 10^9^/L) and white blood cell count (> 10 × 10^9^/L) were set according to the definitions of refractoriness/intolerance to hydroxyurea, with reference to overseas guidelines [12, 19]. It was noted that treatment was rarely changed immediately following an increase in platelet and white blood cell counts that exceeded these reference values. Therefore, in Questionnaire 2, all statements were revised to refer to persistent thrombocytosis/leukocytosis. Although this change slightly increased the agreement rate, consensus was still not achieved. The lack of clear reference values and clinical evidence may explain why consensus was not reached.

For patients with high-risk PV initiating cytoreductive therapy, the expert panelists reached consensus regarding the use of ropeginterferon alfa-2b as a first-line cytoreductive treatment in high-risk PV patients aged ≤ 70 years. This likely reflects the growing confidence of clinicians in treating older patients with ropeginterferon alfa-2b based on tolerance and other factors, and suggests that ≤ 70 years is an appropriate age criterion for the use of ropeginterferon alfa-2b. An overseas clinical trial that compared ropeginterferon alfa-2b with standard therapy in patients with PV reported that ropeginterferon alfa-2b treatment reduced the *JAK2* V617F allele burden and improved the event-free survival [14]. Those findings may have encouraged the panelists to agree that patients aged ≤ 70 years with controlled hematocrit (< 45%) who had already been treated with hydroxyurea should be switched to ropeginterferon alfa-2b.

In the current Japanese guideline, ropeginterferon alfa-2b was included as a first-line cytoreductive therapy for patients with high-risk PV, and for the patients currently treated with hydroxyurea, ruxolitinib is recommended [6]. In this study, for patients with high-risk PV already undergoing treatment with hydroxyurea, ropeginterferon alfa-2b was suggested by the expert panelists as an alternative treatment when standard therapies prove insufficient or intolerant. These patients should be switched to ropeginterferon alfa-2b if they experience hydroxyurea-related toxicity, persistent PV-related symptoms, persistently elevated hematocrit, persistent leukocytosis or thrombocytosis, or poor medication adherence with poor blood cell control. International guidelines recommend ruxolitinib and ropeginterferon alfa-2b for patients with high-risk PV who respond inadequately to hydroxyurea [12], and the results of this expert consensus suggest that ropeginterferon alfa-2b may also be considered as an option for these patients.

Splenomegaly emerged as a unique challenge across the low- and high-risk populations, and no consensus was reached regarding the use of ropeginterferon alfa-2b in patients with low- and high-risk PV based on the presence of splenomegaly alone. For patients with low-risk PV treated with phlebotomy and/or aspirin who develop splenomegaly (without progression to myelofibrosis), the panelists agreed with the initiation of cytoreductive therapy. For hydroxyurea-treated patients with low- and high-risk PV, in cases where splenomegaly (without progression to myelofibrosis) does not reduce, consensus was reached regarding switching to another cytoreductive therapy. However, consensus was not reached regarding the use of ropeginterferon alfa-2b as the first-line cytoreductive therapy in these patients. This lack of agreement may be based on the results of the RESPONSE study [20, 21], suggesting that patients with low- or high-risk PV will most likely be treated with ruxolitinib.

No consensus was reached on statements regarding the role of the *JAK2* V617F allele burden in treatment decisions, such as whether ropeginterferon alfa-2b could be initiated in low-risk patients with a high *JAK2* V617F allele burden (> 50%) at diagnosis. Currently, there is insufficient evidence to support the use of the *JAK2* V617F allele burden as a treatment target, and it is not routinely measured in clinical practice during follow-up. Moreover, for patients who are assessed for the *JAK2* V617F allele burden, ongoing monitoring of allele burden is seldom performed because of its cost and the lack of insurance coverage. Additionally, although some studies have provided evidence that ropeginterferon alfa-2b may contribute to a reduction in the *JAK2* V617F allele burden [10, 22, 23], we speculate that many clinicians prioritize thrombosis prevention as the primary goal of PV treatment, and may not consider the molecular response.

There was strong consensus that ropeginterferon alfa-2b is an appropriate cytoreductive therapy for patients who are pregnant or are planning to conceive. It is notable that consensus was reached on these statements considering the characteristics of the experts surveyed. This consistency across the panelists could be clinically significant considering the general complexity and cautious approach required in treating pregnant patients. The guidelines for cancer treatment in the United States recommend the use of peginterferon alfa-2a for patients who are pregnant and require cytoreductive therapy. However, other pegylated interferons, such as ropeginterferon alfa-2b, are appropriate if peginterferon alfa-2a is unavailable. Additionally, several case studies have described the outcomes following the use of ropeginterferon alfa-2b in three pregnant patients with PV, and both the patients and their babies had no safety issues [24–26]. However, there is currently no robust evidence regarding the use of ropeginterferon alfa-2b during pregnancy or in patients wishing to conceive. Therefore, its use should only be cautiously considered after careful evaluation of the individual patient’s characteristics.

As this study was performed using the Delphi method, which ensures anonymity, each panelist could express independent opinions. However, this study does have some limitations. First, ropeginterferon alfa-2b had only been recently introduced in Japan; thus, there was limited experience of the use of this drug in clinical practice. Second, evidence regarding the use of this drug in each of the patient groups was limited. Third, consensus was based on the Likert scale results, and the rationales for agreement/non-agreement were not collected or analyzed; therefore, revisions to the statements included in Questionnaire 2 were made by the Steering Committee based on their knowledge and experience. Consensus was not reached for some statements after two rounds of review. Further rounds may have been required to provide sufficient opportunity to revise or evolve those statements, or to assess why consensus was not reached. Fourth, although the Delphi method is designed to support independent thinking and iterative consensus-building through anonymization, we acknowledge that it is possible that the presentation of aggregated results from Questionnaire 1 may have influenced individual judgments in Questionnaire 2. Fifth, consensus was achieved regarding the use of ropeginterferon alfa-2b when initiating or switching cytoreductive therapy, but continuation of treatment or the use of other drugs was outside the scope of this study. Finally, bias-related safeguards including anonymization, a multi-round approach, the disclosure of study sponsorship in the consent form, and lack of financial incentives for panel members were incorporated into the design of the study. Nevertheless, the possibility of bias should be acknowledged. Although the sponsor was not involved in data collection, analysis, or interpretation, the risk of sponsorship bias cannot be entirely excluded because of their involvement in the study’s early-stage planning. Furthermore, all Steering Committee members have past or current financial relationships with the sponsor, which may also have introduced bias in the interpretation or weighting of certain consensus statements. Because PV is a rare disease and there are a limited number of specialists involved in patient care, collecting and disclosing individual conflicts of interest could have risked compromising participant anonymity. Moreover, excluding potential panelists based on their conflicts of interest could have made it difficult to establish a sufficiently qualified panel. For these reasons, conflicts of interest were not formally confirmed or used as an exclusion criterion in this study. Therefore, careful interpretation of these findings is warranted and emphasize the importance of selecting treatment according to the individual characteristics of each patient. Nevertheless, the Japanese guidelines suggest ropeginterferon alfa-2b as a first-line cytoreductive therapy in patients with low-risk PV treated with phlebotomy and/or aspirin who have difficulty in continuing phlebotomy or who have markedly elevated platelet or white blood cell counts [6], and the results of this expert consensus were consistent with these guidelines. Furthermore, this study revealed consensus regarding other scenarios in which the use of ropeginterferon alfa-2b may be appropriate. As clinical experience with ropeginterferon alfa-2b accumulates, it is likely that recommendations may change, and reevaluation may be necessary as new clinical data emerge.

## Conclusion

This study used the Delphi method to gain expert consensus regarding which patient groups should be treated with ropeginterferon alfa-2b, and when patients should be switched from other treatments to ropeginterferon alfa-2b. This expert consensus contributes to the appropriate selection of patients for this drug in clinical practice. In patients with risk factors other than splenomegaly, experts tend to favor ropeginterferon alfa-2b as the first-line treatment.

## Supplementary Information

Below is the link to the electronic supplementary material.

Supplementary file1 (PDF 235 KB)

## Data Availability

All data supporting the findings of this study are available within the paper and its Supplementary Information.
