# Supplementary material for: Consensus on the use of ropeginterferon alfa-2b in Japanese patients with polycythemia vera: a modified Delphi survey
Source: Int J Hematol. 2026 Mar 4;123(5):664–77. doi: 10.1007/s12185-025-04140-8 (PMC13172012; doi:10.1007/s12185-025-04140-8)
Supplement: Supplementary file 1 — Supplementary file1 (PDF 235 KB) [file 12185_2025_4140_MOESM1_ESM.pdf]

Supplementary Table 1. Survey questions and outcomes.

Statement No.	Questionnaire 1	Questionnaire 2	Agreement in Questionnaire 1, % (n/N)	Agreement in Questionnaire 2, % (n/N)	Outcome
**Low-risk PV: Patients being treated with phlebotomy and/or aspirin (16 statements)**					
1-1	In low-risk PV patients treated with a Ht target of <45% with phlebotomy, if syncope or blood phobia is observed following treatment with phlebotomy, or if intravenous access is difficult, initiate cytoreductive therapy	No change from Questionnaire 1	94.4 (17/18)	94.4 (17/18)	Consensus achieved
1-2	For initiating cytoreductive therapy in these patients, ropeginterferon alfa-2b is a first-line treatment	No change from Questionnaire 1	82.4 (14/17*)	82.4 (14/17*)	Consensus achieved
2-1	In low-risk PV patients treated with a Ht target of <45% with phlebotomy, if symptoms suggestive of or associated with iron deficiency (e.g., malaise) are observed, initiate cytoreductive therapy	In low-risk PV patients treated with a Ht target of <45%, if symptoms associated with iron deficiency (e.g., malaise) persist after treatment with phlebotomy, initiate cytoreductive therapy	72.2 (13/18)	94.4 (17/18)	Consensus achieved
2-2	For initiating cytoreductive therapy in these patients, ropeginterferon alfa-2b is a first-line treatment	No change from Questionnaire 1	76.9 (10/13*)	82.4 (14/17*)	Consensus achieved
3-1	In low-risk PV patients treated with a Ht target of <45% with phlebotomy, if a patient feels pain resulting from phlebotomy, initiate cytoreductive therapy	No change from Questionnaire 1	94.4 (17/18)	94.4 (17/18)	Consensus achieved

Statement No.	Questionnaire 1	Questionnaire 2	Agreement in Questionnaire 1, % (n/N)	Agreement in Questionnaire 2, % (n/N)	Outcome
3-2	For initiating cytoreductive therapy in these patients, ropeginterferon alfa-2b is a first-line treatment	No change from Questionnaire 1	82.4 (14/17*)	82.4 (14/17*)	Consensus achieved
4-1	In low-risk PV patients, if symptoms (e.g., itching, headache, erythromelalgia, or vasomotor symptoms not responsive to aspirin) are not improved by treatment with phlebotomy and/or aspirin, initiate cytoreductive therapy	No change from Questionnaire 1	100.0 (18/18)	94.4 (17/18)	Consensus achieved
4-2	For initiating cytoreductive therapy in these patients, ropeginterferon alfa-2b is a first-line treatment	No change from Questionnaire 1	83.3 (15/18*)	88.2 (15/17*)	Consensus achieved
5-1	In low-risk PV patients, if significant thrombocytosis (platelet count >1,000 × 10^9 /L) <u>is observed</u>, initiate cytoreductive therapy	In low-risk PV patients, if significant thrombocytosis (platelet count >1,000 × 10^9 /L) <u>persists</u>, initiate cytoreductive therapy	77.8 (14/18)	77.8 (14/18)	Consensus achieved
5-2	For initiating cytoreductive therapy in these patients, ropeginterferon alfa-2b is a first-line treatment	No change from Questionnaire 1	92.9 (13/14*)	85.7 (12/14*)	Consensus achieved
6-1	In low-risk PV patients, if leukocytosis (white blood cell count >15 × 10^9/L) <u>is observed</u>, initiate cytoreductive therapy	In low-risk PV patients, if leukocytosis (white blood cell count >15 × 10^9/L) <u>persists</u>, initiate cytoreductive therapy	88.9 (16/18)	88.9 (16/18)	Consensus achieved

Statement No.	Questionnaire 1	Questionnaire 2	Agreement in Questionnaire 1, % (n/N)	Agreement in Questionnaire 2, % (n/N)	Outcome
6-2	For initiating cytoreductive therapy in these patients, ropeginterferon alfa-2b is a first-line treatment	No change from Questionnaire 1	87.5 (14/16*)	87.5 (14/16*)	Consensus achieved
7-1	In low-risk PV patients, if splenomegaly (with confirmation that there is no progression to myelofibrosis) is observed, initiate cytoreductive therapy	No change from Questionnaire 1	77.8 (14/18)	77.8 (14/18)	Consensus achieved
7-2	For initiating cytoreductive therapy in these patients, ropeginterferon alfa-2b is a first-line treatment	No change from Questionnaire 1	71.4 (10/14*)	71.4 (10/14*)	Did not achieve consensus
8	In low-risk PV patients with a high *JAK2* V617F allele burden (>50%) at diagnosis, initiate treatment with ropeginterferon alfa-2b	No change from Questionnaire 1	66.7 (12/18)	66.7 (12/18)	Did not achieve consensus
9	In low-risk PV patients, even if Ht can be controlled (Ht <45% is achieved and maintained) with phlebotomy and/or aspirin, treatment with ropeginterferon alfa-2b is initiated to decrease the *JAK2* V617F allele burden	No change from Questionnaire 1	50.0 (9/18)	50.0 (9/18)	Did not achieve consensus

Statement No.	Questionnaire 1	Questionnaire 2	Agreement in Questionnaire 1, % (n/N)	Agreement in Questionnaire 2, % (n/N)	Outcome
**Low-risk PV: Patients being treated with hydroxyurea (17 statements)**					
10-1	In low-risk PV patients who require treatment with hydroxyurea, if unacceptable non-hematological toxicity (e.g., leg ulcers, mucocutaneous manifestations, gastrointestinal symptoms, pneumonitis, or fever) occurs during treatment with hydroxyurea, treatment is switched to other drugs	No change from Questionnaire 1	100.0 (18/18)	100.0 (18/18)	Consensus achieved
10-2	When treatment is switched to other drugs in these patients, ropeginterferon alfa-2b is a first-line treatment	No change from Questionnaire 1	88.9 (16/18*)	100.0 (18/18*)	Consensus achieved
11-1	In low-risk PV patients, if symptoms (e.g., itching, headache, erythromelalgia, or vasomotor symptoms not responsive to aspirin) are not improved during treatment with hydroxyurea, treatment is switched to other drugs	No change from Questionnaire 1	100.0 (18/18)	100.0 (18/18)	Consensus achieved
11-2	When treatment is switched to other drugs in these patients, ropeginterferon alfa-2b is a first-line treatment	No change from Questionnaire 1	88.9 (16/18*)	100.0 (18/18*)	Consensus achieved

Statement No.	Questionnaire 1	Questionnaire 2	Agreement in Questionnaire 1, % (n/N)	Agreement in Questionnaire 2, % (n/N)	Outcome
12-1	In low-risk PV patients who require treatment with hydroxyurea, if Ht <45% cannot be achieved or frequent phlebotomy is necessary to achieve Ht <45% during treatment with hydroxyurea at the maximum tolerated dose, treatment is switched to other drugs	No change from Questionnaire 1	94.4 (17/18)	94.4 (17/18)	Consensus achieved
12-2	When treatment is switched to other drugs in these patients, ropeginterferon alfa-2b is a first-line treatment	No change from Questionnaire 1	82.4 (14/17*)	100.0 (17/17*)	Consensus achieved
13-1	In low-risk PV patients who require treatment with hydroxyurea, if thrombocytosis (platelet count >400 × 10^9/L) is observed during treatment with hydroxyurea at the maximum tolerated dose, treatment is switched to other drugs	In low-risk PV patients who require treatment with hydroxyurea, if thrombocytosis (platelet count >400 × 10^9/L) persists during treatment with hydroxyurea at the maximum tolerated dose, treatment is switched to other drugs	50.0 (9/18)	61.1 (11/18)	Did not achieve consensus
13-2	When treatment is switched to other drugs in these patients, ropeginterferon alfa-2b is a first-line treatment	No change from Questionnaire 1	100 (9/9*)	100 (11/11*)	Reference data

Statement No.	Questionnaire 1	Questionnaire 2	Agreement in Questionnaire 1, % (n/N)	Agreement in Questionnaire 2, % (n/N)	Outcome
14-1	In low-risk PV patients who require treatment with hydroxyurea, if leukocytosis (white blood cell count >10 × 10^9 cells/L) is observed during treatment with hydroxyurea at the maximum tolerated dose, treatment is switched to other drugs	In low-risk PV patients who require treatment with hydroxyurea, if leukocytosis (white blood cell count >10 × 10^9/L) persists during treatment with hydroxyurea at the maximum tolerated dose, treatment is switched to other drugs	66.7 (12/18)	72.2 (13/18)	Did not achieve consensus
14-2	When treatment is switched to other drugs in these patients, ropeginterferon alfa-2b is a first-line treatment	No change from Questionnaire 1	91.7 (11/12*)	92.3 (12/13*)	Reference data
15-1	In low-risk PV patients who require treatment with hydroxyurea, if a reduction in splenomegaly (with confirmation that there is no progression to myelofibrosis) is not observed during treatment with hydroxyurea at the maximum tolerated dose, treatment is switched to other drugs	No change from Questionnaire 1	77.8 (14/18)	88.9 (16/18)	Consensus achieved
15-2	When treatment is switched to other drugs in these patients, ropeginterferon alfa-2b is a first-line treatment	No change from Questionnaire 1	71.4 (10/14*)	62.5 (10/16*)	Did not achieve consensus

Statement No.	Questionnaire 1	Questionnaire 2	Agreement in Questionnaire 1, % (n/N)	Agreement in Questionnaire 2, % (n/N)	Outcome
16-1	In low-risk PV patients who require treatment with hydroxyurea, if a decrease in the *JAK2* V617F allele burden is not observed during treatment with hydroxyurea at the maximum tolerated dose, treatment is switched to other drugs	No change from Questionnaire 1	66.7 (12/18)	61.1 (11/18)	Did not achieve consensus
16-2	When treatment is switched to other drugs in these patients, ropeginterferon alfa-2b is a first-line treatment	No change from Questionnaire 1	91.7 (11/12*)	90.9 (10/11*)	Reference data
17	In low-risk PV patients who require treatment with hydroxyurea, if medication adherence <u>is poor</u> during treatment with hydroxyurea, treatment is switched to ropeginterferon alfa-2b	In low-risk PV patients who require treatment with hydroxyurea, if medication adherence <u>is poor and blood cells are uncontrolled</u> during treatment with hydroxyurea, treatment is switched to ropeginterferon alfa-2b	38.9 (7/18)	61.1 (11/18)	Did not achieve consensus
18	In low-risk PV patients who require cytoreductive therapy and are treated with hydroxyurea, treatment is switched to ropeginterferon alfa-2b considering the risk of secondary leukemia	No change from Questionnaire 1	72.2 (13/18)	66.7 (12/18)	Did not achieve consensus

Statement No.	Questionnaire 1	Questionnaire 2	Agreement in Questionnaire 1, % (n/N)	Agreement in Questionnaire 2, % (n/N)	Outcome
19	In low-risk PV patients who require treatment with hydroxyurea, even if Ht can be controlled (Ht <45% is achieved and maintained) during treatment with hydroxyurea, treatment is switched to ropeginterferon alfa-2b to decrease the *JAK2* V617F allele burden	No change from Questionnaire 1	61.1 (11/18)	55.6 (10/18)	Did not achieve consensus
**High-risk PV: Patients about to initiate cytoreductive therapy (8 statements)**					
20	In the following high-risk PV patients who are considered to require cytoreductive therapy, ropeginterferon alfa-2b is used as a first-line treatment	No change from Questionnaire 1	-	-	-
20-1	High-risk PV patients aged <60 years with a history of thrombosis	No change from Questionnaire 1	100.0 (18/18)	100.0 (18/18)	Consensus achieved
20-2	High-risk PV patients aged ≥60 years and ≤65 years	No change from Questionnaire 1	83.3 (15/18)	94.4 (17/18)	Consensus achieved
20-3	High-risk PV patients aged ≥66 years and ≤70 years	No change from Questionnaire 1	66.7 (12/18)	77.8 (14/18)	Consensus achieved
20-4	High-risk PV patients aged >70 years	No change from Questionnaire 1	38.9 (7/18)	61.1 (11/18)	Did not achieve consensus

Statement No.	Questionnaire 1	Questionnaire 2	Agreement in Questionnaire 1, % (n/N)	Agreement in Questionnaire 2, % (n/N)	Outcome
21	In the following high-risk PV patients with *JAK2* V617F allele burden >50% at diagnosis and who are considered to require cytoreductive therapy, ropeginterferon alfa-2b is used as a first-line treatment:	No change from Questionnaire 1	-	-	-
21-1	High-risk PV patients aged <60 years with a history of thrombosis	No change from Questionnaire 1	94.4 (17/18)	100.0 (18/18)	Consensus achieved
21-2	High-risk PV patients aged ≥60 years and ≤65 years	No change from Questionnaire 1	83.3 (15/18)	94.4 (17/18)	Consensus achieved
21-3	High-risk PV patients aged ≥66 years and ≤70 years	No change from Questionnaire 1	72.2 (13/18)	77.8 (14/18)	Consensus achieved
21-4	High-risk PV patients aged >70 years	No change from Questionnaire 1	50.0 (9/18)	61.1 (11/18)	Did not achieve consensus
**High-risk PV: Patients being treated with hydroxyurea or ruxolitinib (23 statements)**					
22-1	In high-risk PV patients, if unacceptable non-hematological toxicity (e.g., leg ulcers, mucocutaneous manifestations, gastrointestinal symptoms, pneumonitis, or fever) occurs during treatment with hydroxyurea, treatment is switched to other drugs	No change from Questionnaire 1	100.0 (18/18)	100.0 (18/18)	Consensus achieved

Statement No.	Questionnaire 1	Questionnaire 2	Agreement in Questionnaire 1, % (n/N)	Agreement in Questionnaire 2, % (n/N)	Outcome
22-2	When treatment is switched to other drugs in these patients, ropeginterferon alfa-2b is a first-line treatment	No change from Questionnaire 1	88.9 (16/18*)	94.4 (17/18*)	Consensus achieved
23-1	In high-risk PV patients, if symptoms (e.g., itching, headache, erythromelalgia, or vasomotor symptoms that are not responsive to aspirin) are not improved during treatment with hydroxyurea, treatment is switched to other drugs	No change from Questionnaire 1	100.0 (18/18)	100.0 (18/18)	Consensus achieved
23-2	When treatment is switched to other drugs in these patients, ropeginterferon alfa-2b is a first-line treatment	No change from Questionnaire 1	83.3 (15/18*)	88.9 (16/18*)	Consensus achieved
24-1	In high-risk PV patients, if Ht <45% cannot be achieved or if frequent phlebotomy is necessary to achieve Ht <45% during treatment with hydroxyurea at the maximum tolerated dose, treatment is switched to other drugs	No change from Questionnaire 1	100.0 (18/18)	100.0 (18/18)	Consensus achieved
24-2	When treatment is switched to other drugs in these patients, ropeginterferon alfa-2b is a first-line treatment	No change from Questionnaire 1	77.8 (14/18*)	88.9 (16/18*)	Consensus achieved

Statement No.	Questionnaire 1	Questionnaire 2	Agreement in Questionnaire 1, % (n/N)	Agreement in Questionnaire 2, % (n/N)	Outcome
25-1	In high-risk PV patients, if thrombocytosis (platelet count >400 × 10^9/L) is observed during treatment with hydroxyurea at the maximum tolerated dose, treatment is switched to other drugs	In high-risk PV patients, if thrombocytosis (platelet count >400 × 10^9/L) persists during treatment with hydroxyurea at the maximum tolerated dose, treatment is switched to other drugs	77.8 (14/18)	88.9 (16/18)	Consensus achieved
25-2	When treatment is switched to other drugs in these patients, ropeginterferon alfa-2b is a first-line treatment	No change from Questionnaire 1	85.7 (12/14*)	100.0 (16/16*)	Consensus achieved
26-1	In high-risk PV patients, if leukocytosis (white blood cell count >10 × 10^9/L) is observed during treatment with hydroxyurea at the maximum tolerated dose, treatment is switched to other drugs	In high-risk PV patients, if leukocytosis (white blood cell count >10 × 10^9/L) persists during treatment with hydroxyurea at the maximum tolerated dose, treatment is switched to other drugs	88.9 (16/18)	94.4 (17/18)	Consensus achieved
26-2	When treatment is switched to other drugs in these patients, ropeginterferon alfa-2b is a first-line treatment	No change from Questionnaire 1	75.0 (12/16*)	94.1 (16/17*)	Consensus achieved

Statement No.	Questionnaire 1	Questionnaire 2	Agreement in Questionnaire 1, % (n/N)	Agreement in Questionnaire 2, % (n/N)	Outcome
27-1	In high-risk PV patients, if a reduction in splenomegaly (with confirmation that there is no progression to myelofibrosis) is not observed during treatment with hydroxyurea at the maximum tolerated dose, treatment is switched to other drugs	No change from Questionnaire 1	88.9 (16/18)	94.4 (17/18)	Consensus achieved
27-2	When treatment is switched to other drugs in these patients, ropeginterferon alfa-2b is a first-line treatment	No change from Questionnaire 1	68.8 (11/16*)	70.6 (12/17*)	Did not achieve consensus
28-1	In high-risk PV patients, if a decrease in the *JAK2* V617F allele burden is not observed during treatment with hydroxyurea, treatment is switched to other drugs	No change from Questionnaire 1	72.2 (13/18)	55.6 (10/18)	Did not achieve consensus
28-2	When treatment is switched to other drugs in these patients, ropeginterferon alfa-2b is a first-line treatment	No change from Questionnaire 1	84.6 (11/13*)	100.0 (10/10*)	Reference data
29	In high-risk PV patients, if medication adherence <u>is poor</u> during treatment with hydroxyurea or ruxolitinib, treatment is switched to ropeginterferon alfa-2b	In high-risk PV patients, if medication adherence <u>is poor and blood cells are uncontrolled</u> during treatment with hydroxyurea or ruxolitinib, treatment is switched to ropeginterferon alfa-2b	61.1 (11/18)	77.8 (14/18)	Consensus achieved

Statement No.	Questionnaire 1	Questionnaire 2	Agreement in Questionnaire 1, % (n/N)	Agreement in Questionnaire 2, % (n/N)	Outcome
30	In the following high-risk PV patients, even if Ht can be controlled (Ht <45% is achieved and maintained) during treatment with hydroxyurea, treatment is switched to ropeginterferon alfa-2b based on a report showing that a decrease in *JAK2* V617F allele burden leads to an improvement in event-free survival	No change from Questionnaire 1	-	-	-
30-1	High-risk PV patients aged <60 years with a history of thrombosis	No change from Questionnaire 1	94.4 (17/18)	94.4 (17/18)	Consensus achieved
30-2	High-risk PV patients aged ≥60 years and ≤65 years	No change from Questionnaire 1	83.3 (15/18)	88.9 (16/18)	Consensus achieved
30-3	High-risk PV patients aged ≥66 years and ≤70 years	No change from Questionnaire 1	66.7 (12/18)	77.8 (14/18)	Consensus achieved
30-4	High-risk PV patients aged >70 years	No change from Questionnaire 1	55.6 (10/18)	50.0 (9/18)	Did not achieve consensus

Statement No.	Questionnaire 1	Questionnaire 2	Agreement in Questionnaire 1, % (n/N)	Agreement in Questionnaire 2, % (n/N)	Outcome
31	In the following high-risk PV patients, even if Ht can be controlled (Ht <45% is achieved and maintained) during treatment with ruxolitinib, treatment is switched to ropeginterferon alfa-2b based on a report showing that a decrease in the *JAK2* V617F allele burden leads to an improvement in event-free survival	No change from Questionnaire 1	-	-	-
31-1	High-risk PV patients aged <60 years with a history of thrombosis	No change from Questionnaire 1	72.2 (13/18)	66.7 (12/18)	Did not achieve consensus
31-2	High-risk PV patients aged ≥60 years and ≤65 years	No change from Questionnaire 1	66.7 (12/18)	66.7 (12/18)	Did not achieve consensus
31-3	High-risk PV patients aged ≥66 years and ≤70 years	No change from Questionnaire 1	55.6 (10/18)	61.1 (11/18)	Did not achieve consensus
31-4	High-risk PV patients aged >70 years	No change from Questionnaire 1	50.0 (9/18)	55.6 (10/18)	Did not achieve consensus

Statement No.	Questionnaire 1	Questionnaire 2	Agreement in Questionnaire 1, % (n/N)	Agreement in Questionnaire 2, % (n/N)	Outcome
**PV patients who are pregnant or planning to conceive (2 statements)**					
32	In PV patients who wish to conceive and require cytoreductive therapy, the use of ropeginterferon alfa-2b is considered	No change from Questionnaire 1	100.0 (18/18)	100.0 (18/18)	Consensus achieved
33	In pregnant PV patients who require cytoreductive therapy, the use of ropeginterferon alfa-2b is considered	No change from Questionnaire 1	100.0 (18/18)	100.0 (18/18)	Consensus achieved

Changes in statements between Questionnaire 1 and Questionnaire 2 are indicated with underlined text.

*For linked statements, the denominator of the following statement was defined as the number of panelists who agreed with the preceding statement.

Ht hematocrit, *JAK2* Janus kinase 2, *PV* polycythemia vera
